# Giant Radicular Cyst of the Pediatric Anterior Maxilla: A Rare Case

**DOI:** 10.7759/cureus.107941

**Published:** 2026-04-29

**Authors:** Supraja Vemuganti, Aishwarya Ravikumar, Ajmera Prem Sagar, Gundlapally Anusha Reddy, Arshiya Sanober, Srimounica Kocherlakota

**Affiliations:** 1 Oral and Maxillofacial Surgery, Government Dental College and Hospital, Hyderabad, IND; 2 Oral and Maxillofacial Surgery, Panineeya Mahavidyalaya Institute of Dental Sciences, Hyderabad, IND

**Keywords:** inflammatory odontogenic cyst, oral and maxillofacial pathology, pediatric oral and maxillofacial surgery, periapical cyst, radicular cyst

## Abstract

Radicular cysts are inflammatory odontogenic cysts that arise from nonvital teeth due to proliferation of the epithelial rests of Malassez. Although they are commonly seen in adults, their occurrence in younger individuals is rare. This case describes a 13-year-old male who presented with pain, swelling, and pus discharge in the left maxillary region, associated with trauma-induced nonvital teeth. Radiographic evaluation revealed a well-defined radiolucent lesion extending into the maxillary sinus with displacement of adjacent structures. The involved teeth were managed with endodontic treatment, followed by surgical enucleation of the lesion and chemical cauterization using 5-fluorouracil. Postoperative healing was uneventful, with preservation of surrounding anatomical structures. This case highlights the consequences of delayed intervention, which led to the formation of a large cyst and subsequent infection. It underscores the importance of timely and effective management of radicular cysts in young patients to prevent extensive structural damage.

## Introduction

A cyst is a pathologic cavity that may or may not be lined by epithelium and is typically filled with fluid, semifluid, or gaseous material, but not pus [1]. A radicular cyst is an inflammatory odontogenic cyst, also referred to as a periapical cyst. These cysts develop from nonvital teeth due to pulp necrosis. The resulting inflammation stimulates the proliferation of the cell rests of Malassez (epithelial remnants of Hertwig’s root sheath) within the periodontal ligament.

Clinically, these cysts may be asymptomatic or may present with acute symptoms such as pain, swelling, occasional pus discharge, and fistula formation when infected. The teeth associated with the lesion may be mobile or displaced and typically fail to respond to electric pulp testing [2]. Radiologically, a periapical cyst is indistinguishable from periapical inflammation, as both appear radiolucent at the apex of tooth roots. Radicular cysts may also arise from lateral canals, in which case they are termed lateral radicular cysts. Root resorption may be observed in some cases.

Management of radicular cysts involves elimination of the source of infection, either through tooth extraction or endodontic treatment, followed by cyst enucleation. These cysts generally do not recur after complete removal. If left untreated, they can progressively destroy surrounding structures, thereby compromising quality of life. Although radicular cysts are more commonly seen in adults, this case highlights their rare occurrence in a young patient. It demonstrates successful management using an appropriate surgical approach that preserved adjacent anatomical structures without complications.

## Case presentation

A 13-year-old male presented to our department with the chief complaint of mild pain, swelling, and pus discharge in the left posterior maxillary region for one month. The patient reported a history of facial trauma three years earlier, resulting in a fracture of the maxillary central incisor (tooth 21).

The patient’s dental history revealed repeated prescriptions of antibiotics (amoxicillin-clavulanic acid (325 mg) and metronidazole (200 mg)) along with the analgesic aceclofenac at a private dental clinic for the management of persistent swelling over a three-month period. There was no relevant medical history.

**Figure 1:**
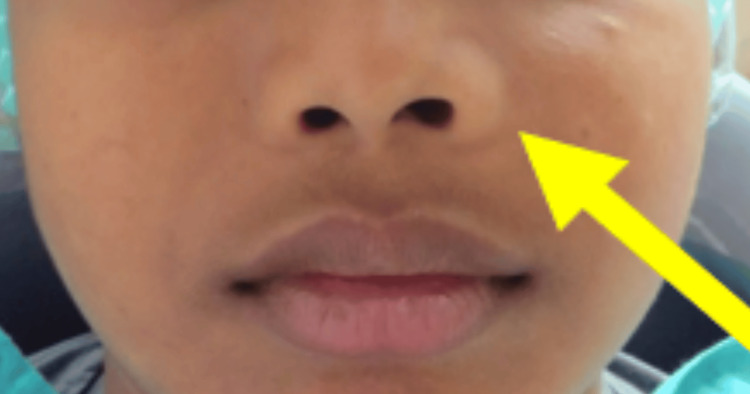
Frontal picture of the patient showing elevation of the ala of the nose and slight swelling on the left middle third of the face depicted by a yellow arrow Written, signed consent from the parent, permitting disclosure of the patient’s identity in an open-access publication, was provided to the journal.

Extraoral examination revealed mild facial asymmetry involving the left middle third of the face, along with slight elevation of the left ala of the nose (Figure 1).

On inspection, the lesion extended approximately two-thirds of the distance from the ala of the nose to the tragus of the ear on the left side. On palpation, it was firm to the touch, with slightly stretched overlying skin and no increase in temperature. The lesion measured approximately 4 × 4 cm.

Intraoral examination revealed a pronounced swelling along the buccal aspect, extending from teeth 21 to 26, as well as palatally, accompanied by pus discharge. An Ellis Class III fracture was noted in relation to tooth 21. The adjacent teeth (22, 23, 24, and 25) were nonvital, as determined by electric pulp testing, showing no response compared with control teeth (12, 13, 14, and 15). On palpation, the intraoral swelling was soft and fluctuant. The palatal mucosa showed no draining sinus tract, and no regional lymphadenopathy was detected. Oral hygiene was fair.

**Figure 2:**
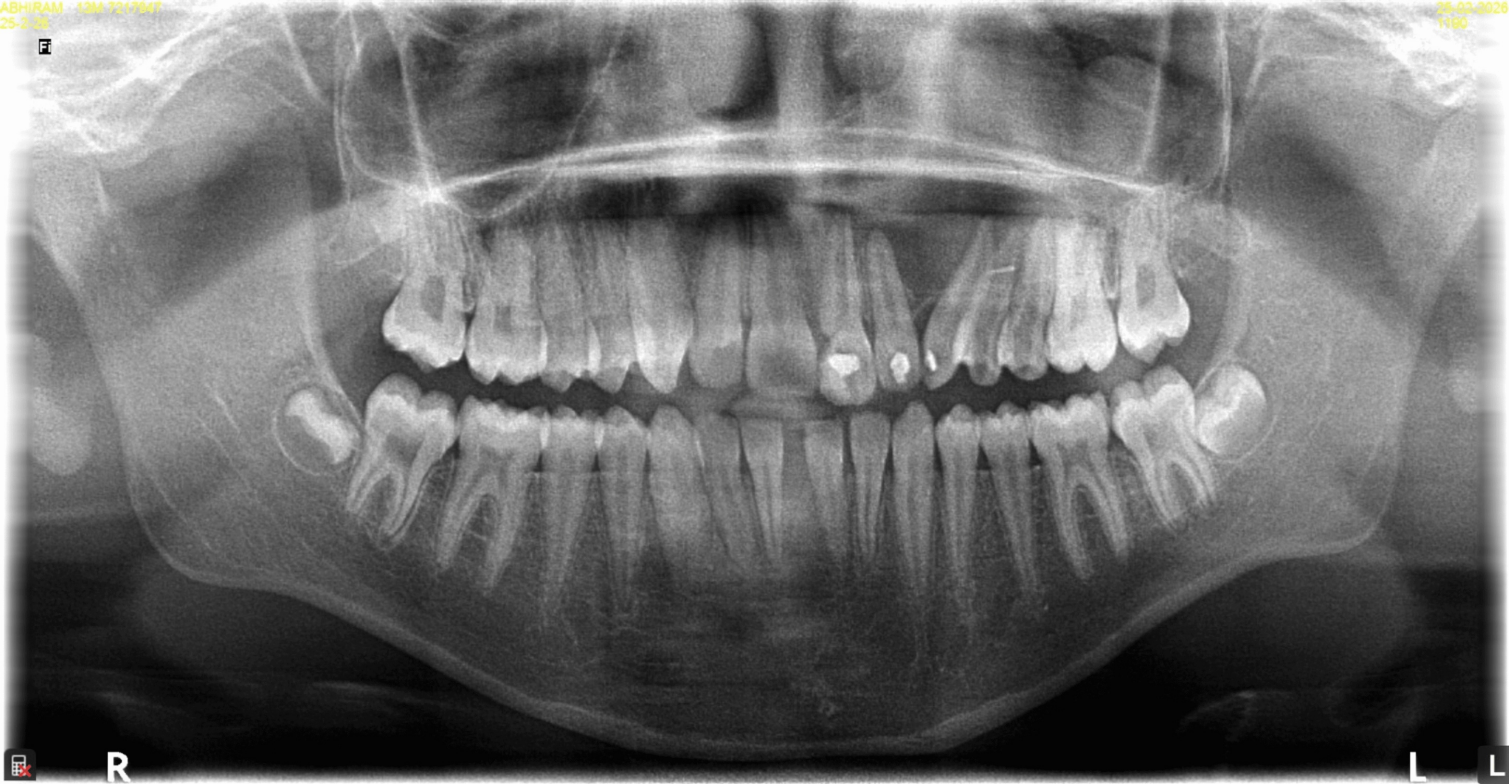
Orthopantomogram showing displaced roots of the left maxillary lateral incisor and left maxillary canine and haziness noted in the left maxillary sinus

The patient presented with a pre-existing orthopantomogram, which revealed a well-defined lesion extending into the left maxillary sinus. The lesion was also seen displacing the roots of the left lateral incisor and canine and extending into the alveolar bone (Figure 2).

**Figure 3:**
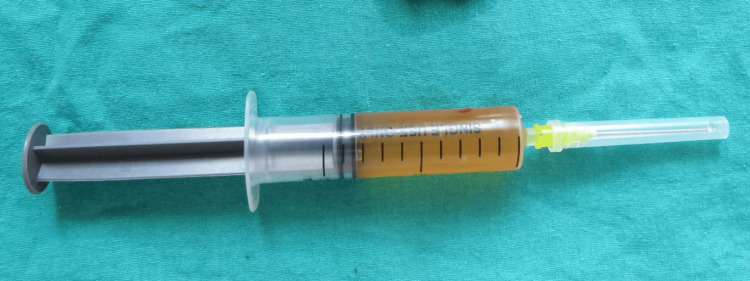
Straw-colored fluid obtained from aspirating the cyst

Further investigation included aspiration of the lesion with a wide-bore needle, yielding approximately 4.5 mL of straw-colored fluid (Figure 3).

**Figure 4:**
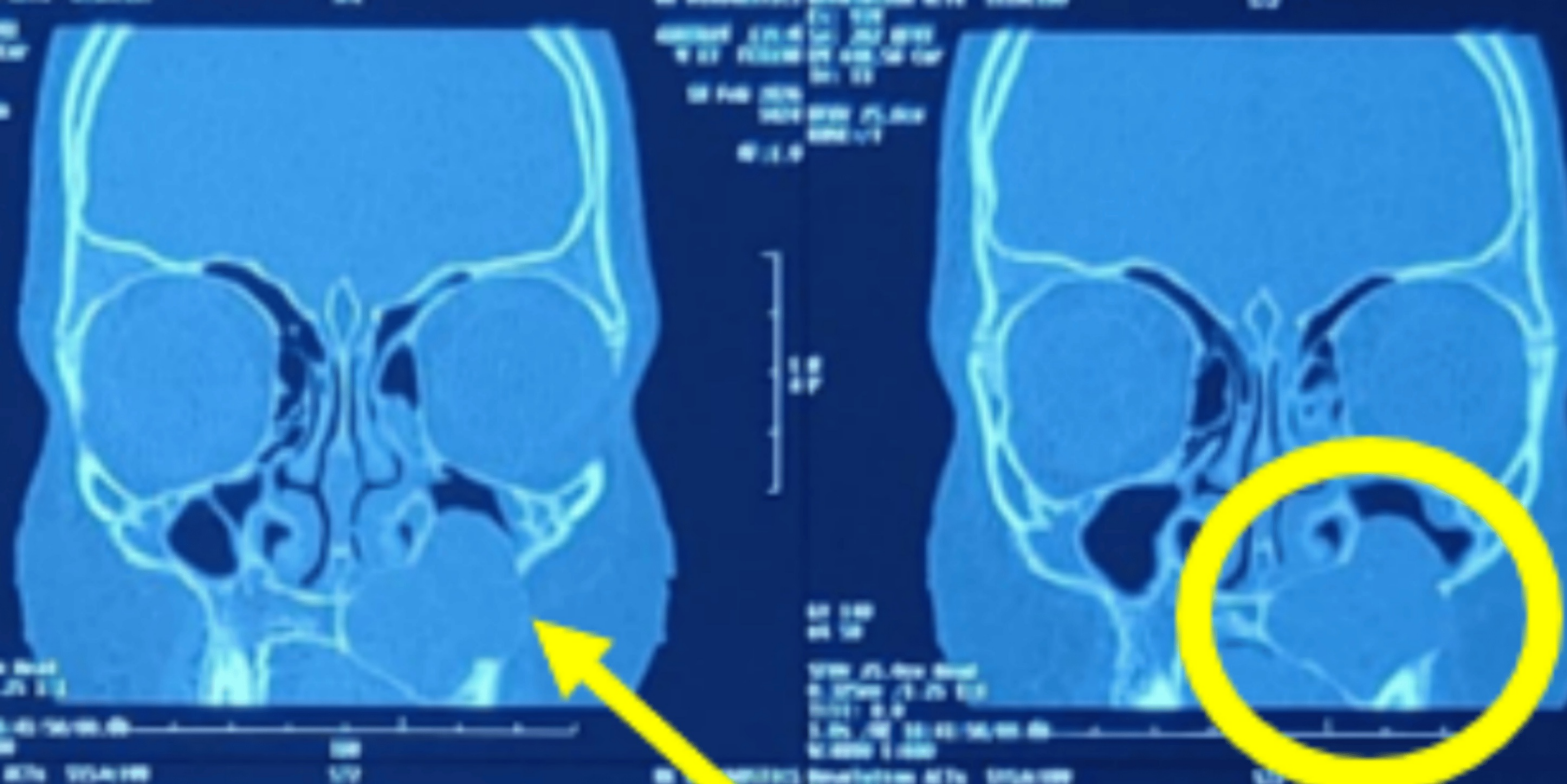
Coronal section of computed tomography showing the extension of the lesion from the mid-palatal region superiorly almost to the roof of the left maxillary sinus, causing perforation in the lateral wall of the sinus

CT was advised to assess the extent of the lesion. Coronal sections revealed a round to ovoid radiolucent lesion with smooth, corticated (well-defined) borders. The lesion was seen to cause a slight elevation of the maxillary sinus floor and nasal floor on the left side, along with thinning of the nasal floor and the floor and lateral wall of the left maxillary sinus (Figure 4).

**Figure 5:**
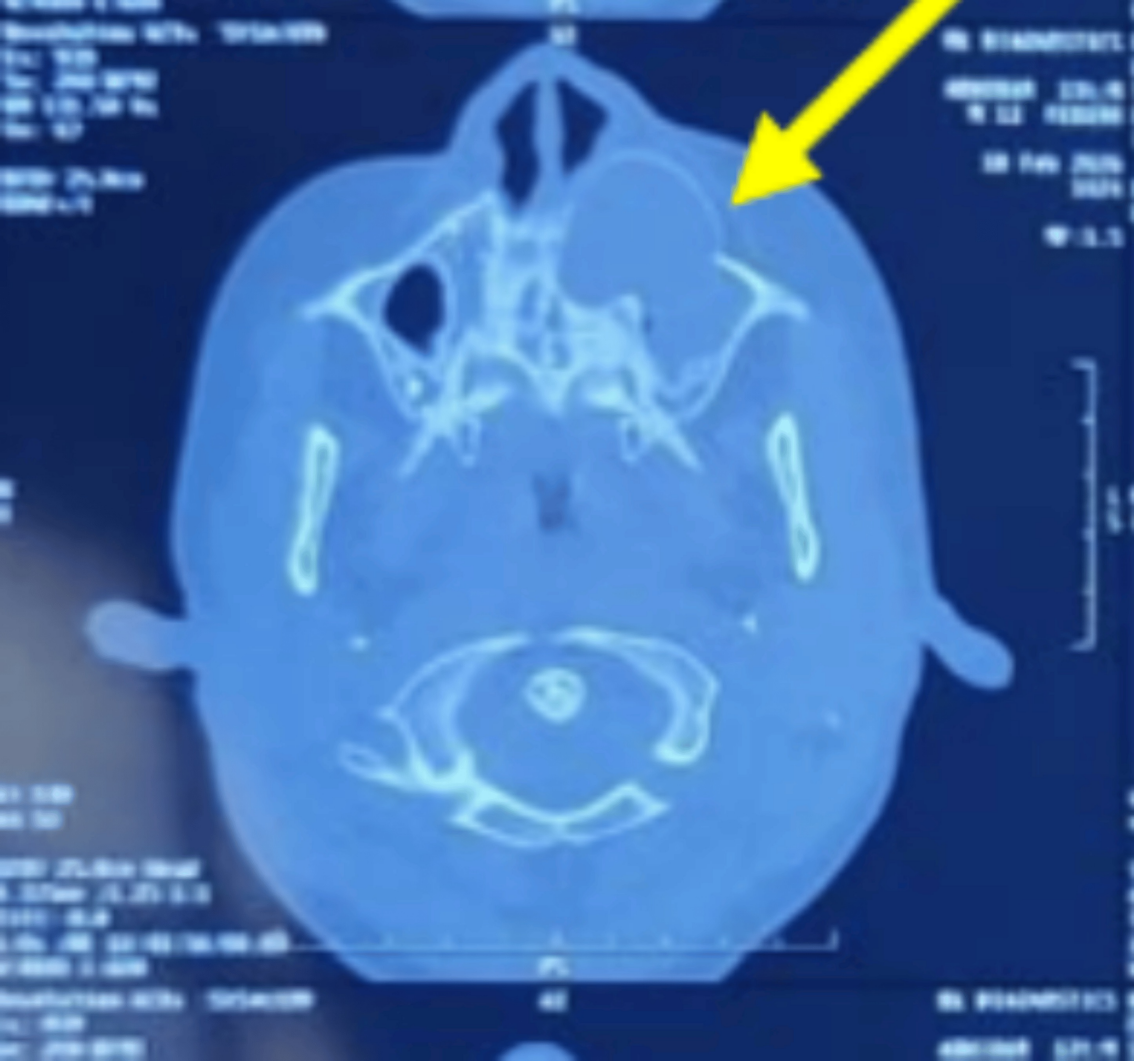
Axial section shows the lesion encroaching on the left maxillary sinus, causing a bulge in the anterior sinus wall and pushing the left lateral wall of the nose

Axial sections demonstrated extension of the lesion into the maxillary alveolus from the midline to the left first molar (26). Thinning and focal breach of the anterior wall of the maxillary sinus were also noted, along with slight displacement of the lateral wall of the nose (Figure 5).

Based on the clinical and radiographic findings, endodontic treatment was performed for teeth 22, 23, 24, and 25, followed by enucleation of the cyst and chemical cauterization using 5-fluorouracil. The procedure was carried out under local anesthesia in sterile, aseptic conditions. Bilateral infraorbital nerve blocks, nasopalatine nerve block, left posterosuperior alveolar nerve block, and left greater palatine nerve block were administered.

**Figure 6:**
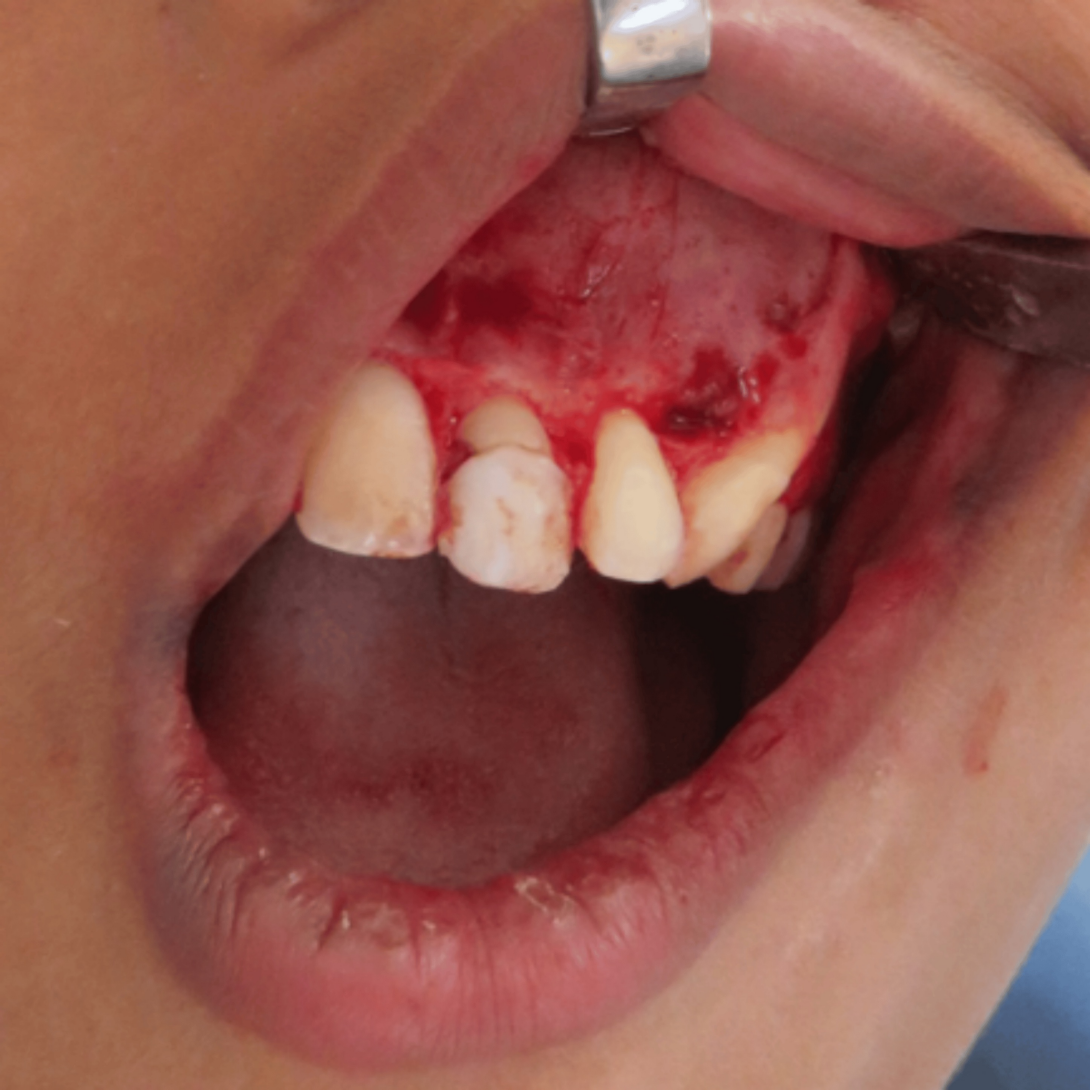
Intraoperative image showing the extent of incision and exposure of the lesion

A crevicular incision was made, and a full-thickness mucoperiosteal flap was raised from the mesial aspect of tooth 12 to the mesial aspect of tooth 26 (Figure 6).

**Figure 7:**
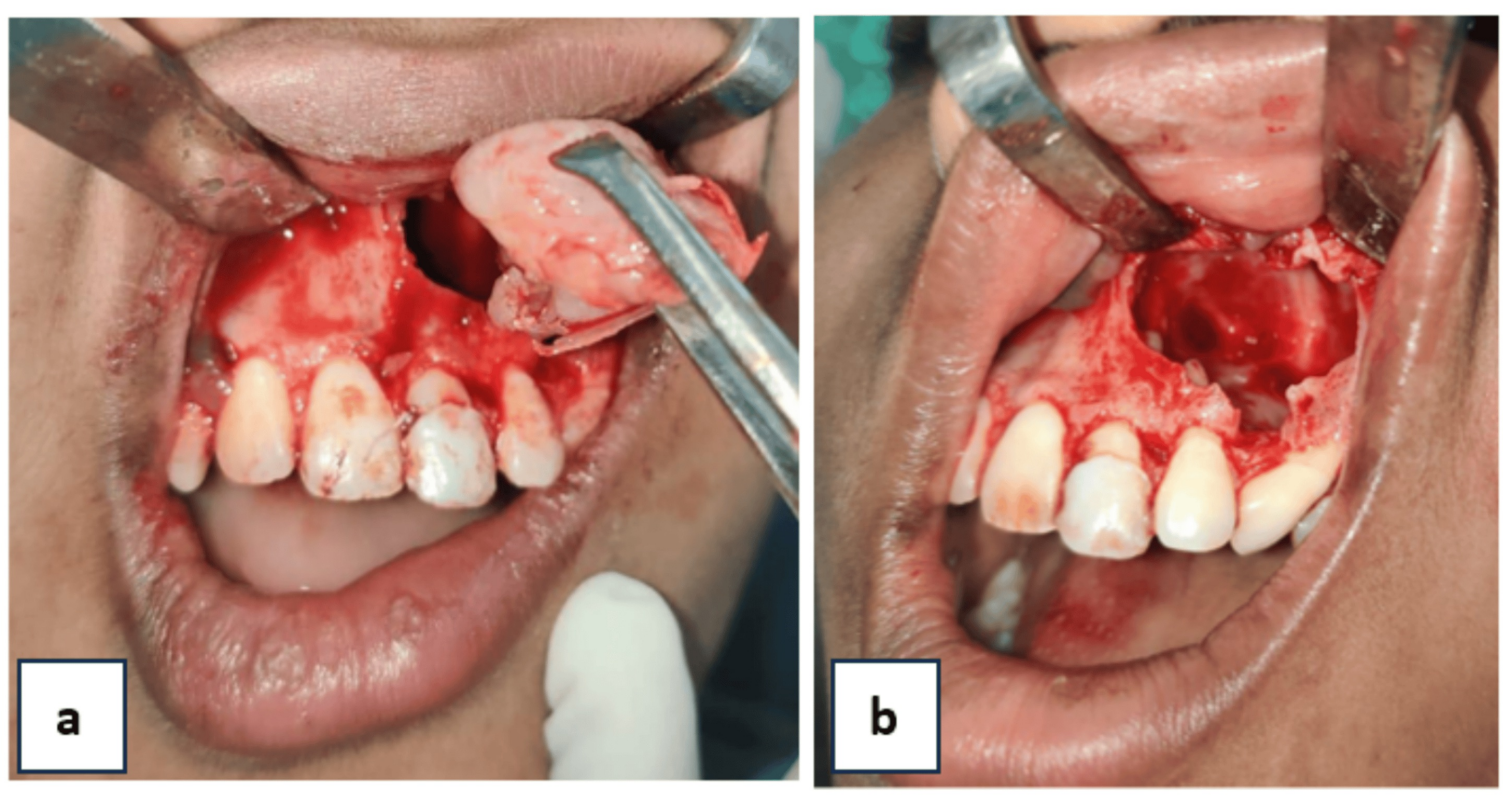
Intraoperative images of cystic lining and cystic cavity (a) enucleated cystic lining, (b) empty cystic cavity

Blunt dissection was performed to separate the cystic lining, which was adherent in certain areas to the labial mucosa. The cystic lining was carefully detached from the walls of the cystic cavity, and complete enucleation was achieved. Subsequently, the cystic cavity was chemically cauterized using 5-fluorouracil (Figure 7).

**Figure 8:**
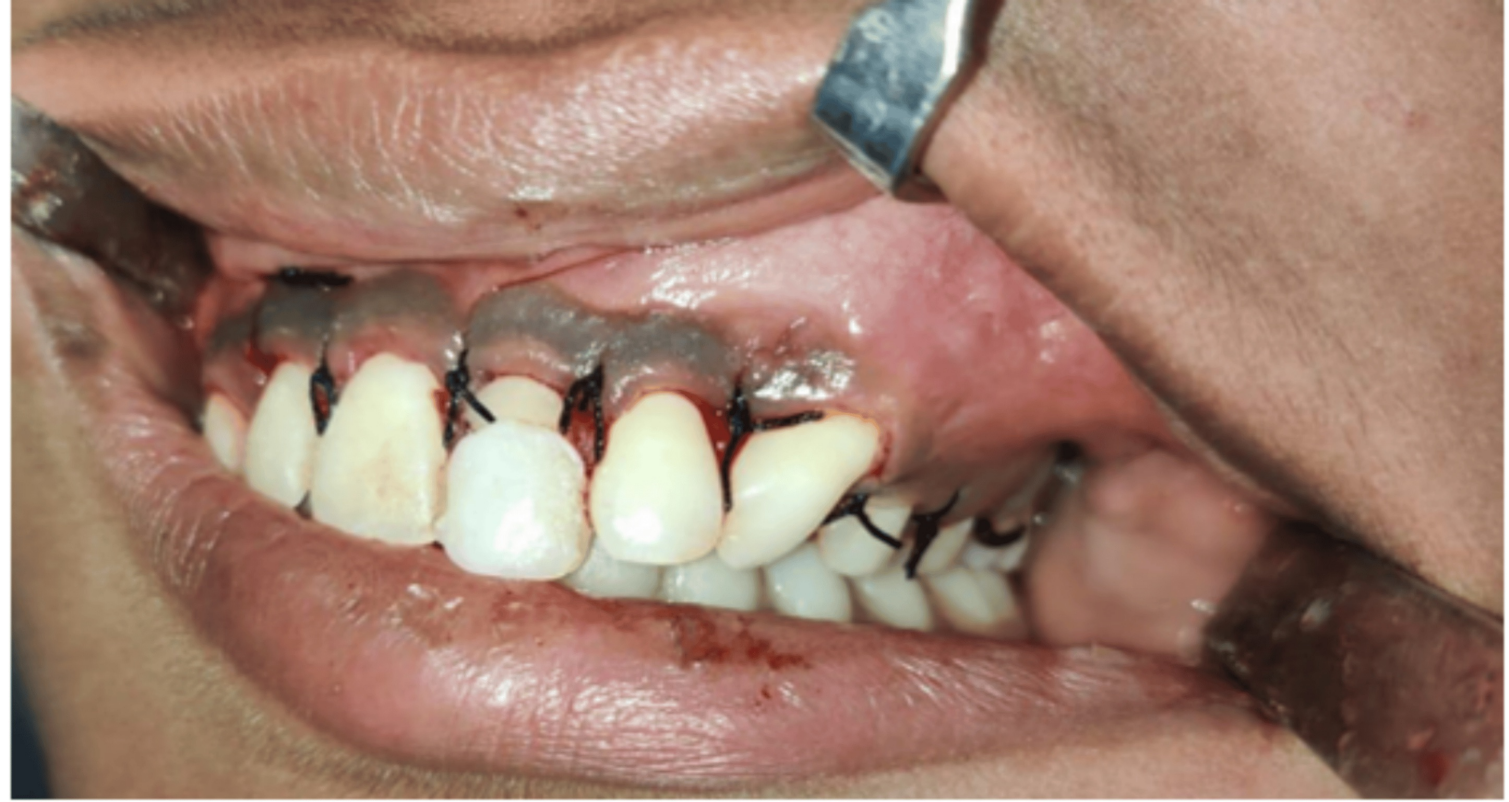
Wound closure using 3-0 braided black silk suture (simple interrupted sutures)

Wound closure was performed using 3-0 black braided silk sutures (Figure 8). An extraoral pressure dressing was applied. The patient was recalled after one week, and postoperative healing was uneventful.

**Figure 9:**
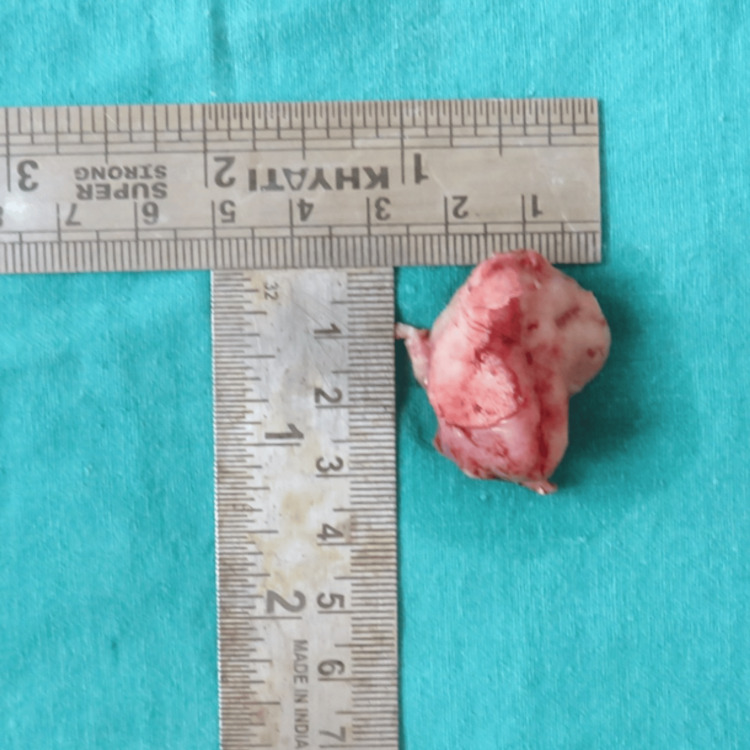
Image of the sample sent to histopathological examination

The cystic lining was sent for histopathological examination (Figure 9). Microscopic examination revealed that the lumen was lined by stratified squamous epithelium of variable thickness exhibiting an arcading pattern. The underlying connective tissue capsule was cellular and showed a dense mixed inflammatory cell infiltrate composed of lymphocytes, neutrophils, and plasma cells. These findings were consistent with an infected radicular cyst.

## Discussion

The radicular cyst is one of the most common odontogenic cysts, typically associated with an infected tooth and subsequent pulp necrosis. Based on its origin, odontogenic cysts may be developmental or inflammatory. In radicular cyst formation, toxins present at the apex of a nonvital tooth induce periapical inflammation, which in turn stimulates the epithelial rests of Malassez located within the periodontal ligament in the apical region. This process leads to the formation of a periapical granuloma, which may be either infected or sterile. Over time, progressive epithelial proliferation and central necrosis due to inadequate blood supply result in the transformation of the granuloma into a cyst. In the present case, a history of trauma three years earlier, resulting in a tooth fracture, likely contributed to pulpal necrosis and subsequent progression to an infected radicular cyst over time [3].

The epithelial rests of Malassez (remnants of Hertwig’s epithelial root sheath), under appropriate stimulation, retain the capacity for epithelial proliferation and are considered to play a key role in the development of radicular cysts.

These cysts occur with the highest incidence in the maxillofacial region [1]. Radicular (or residual) cysts account for up to 60% of all jaw cysts. They may occur in the periapical region of any tooth at any age; however, they are more commonly seen in the third to fifth decades of life, particularly in males, and are rarely associated with primary dentition. This case represents an unusual occurrence of a radicular cyst in a pediatric patient involving permanent dentition. Most periapical cysts are asymptomatic, and the involved teeth are rarely painful or tender to percussion.

In contrast, our patient presented with pain, swelling, and pus discharge, indicating a long-standing infection. Radicular cysts occur more frequently in the maxillary anterior region than in the mandible and are more commonly seen in males, consistent with our case [1]. In the maxilla, swelling typically involves both buccal and palatal aspects, whereas in the mandible it is usually confined to the buccal side and only occasionally extends lingually [4].

Clinically, radicular cysts may mimic other cystic lesions of the oral cavity, such as globulomaxillary cysts, dentigerous cysts, ameloblastoma, odontogenic keratocyst, and odontogenic fibroma, necessitating careful differential diagnosis.

Although radicular cysts are generally slow-growing, they can occasionally reach a size sufficient to cause significant bone destruction, and cortical expansion is relatively uncommon. If left untreated, they progressively enlarge, leading to resorption of adjacent bone. Bone resorption is common, but cortical plate expansion is less frequent compared to lesions such as dentigerous cysts. Patients may avoid extraction of infected anterior teeth, and the porous nature of maxillary bone may facilitate cyst expansion [1]. In some cases, “eggshell cracking” may be elicited due to thinning of the cortical plate. In the present case, the lesion showed both expansion and resorption of the buccal cortical plate, consistent with this finding. As the lesion was long-standing and untreated, it underwent an acute inflammatory exacerbation, resulting in purulent discharge.

Histologically, the cystic cavity is typically lined by non-keratinized stratified squamous epithelium, although areas of dense inflammatory infiltration may alter epithelial characteristics. In early stages, epithelial proliferation may form an arcading pattern associated with active inflammatory infiltration. Rarely, small amounts of keratin may be present.

Management of radicular cysts involving the nasal cavity or maxillary sinus poses a surgical challenge. Conventional radiographs and CBCT alone may not always be sufficient for definitive diagnosis; therefore, histopathological examination remains essential for confirmation [4]. Yalçin et al. reported that radicular cysts are generally oriented perpendicular to the long axis of the bone, have a rounded (globular) shape, and are typically smaller and more uniform in size compared with odontogenic keratocysts (OKCs) [5]. Their classification of lesions based on bony boundaries and potential communication with the nasal floor and maxillary sinus includes five categories, with the present lesion corresponding to Group B [4].

Management of radicular cysts includes extraction of the involved tooth with curettage of periapical tissues or endodontic therapy with apicoectomy and enucleation of the cystic lesion. Considering the patient’s age, conservative management with root canal therapy, enucleation, and chemical cauterization of the cystic cavity using 5-fluorouracil was chosen. Incomplete removal of cystic epithelium may result in residual cyst formation; hence, chemical cauterization was performed to reduce the risk of recurrence.

Recent advances in the management of radicular cysts include the use of curcumin gel, which has anti-inflammatory, antioxidant, and antimicrobial properties that may enhance healing [6]. Other regenerative approaches include the use of resorbable type I bovine collagen membranes with bovine xenografts to promote guided tissue regeneration [7]. Additionally, Jiing-Huei Zhao et al. reported successful use of platelet-rich fibrin and bioactive glass in the management of radicular cysts, demonstrating regenerative potential in cystic bone defects [8].

## Conclusions

Our case report highlights the uncommon presentation of a large radicular cyst involving the anterior maxilla in a pediatric patient. It also underscores how neglect and delayed definitive treatment can lead to cyst formation and progressive expansion, resulting in the destruction of surrounding structures. Early diagnosis through appropriate clinical and radiographic evaluation is critical to prevent extensive tissue damage and associated complications. This case emphasizes the importance of timely management of traumatic dental injuries, avoidance of repeated symptomatic treatment with antibiotics, and adoption of definitive treatment strategies.

The successful management in this case, combining endodontic therapy, complete surgical enucleation, and adjunctive chemical cauterization with 5-fluorouracil, resulted in favorable healing while preserving the surrounding vital structures. Regular follow-up is essential to monitor healing and to detect any recurrence at an early stage.
